# Impaired renal transporter gene expression and uremic toxin excretion as aging hallmarks in cats with naturally occurring chronic kidney disease

**DOI:** 10.18632/aging.206176

**Published:** 2024-12-20

**Authors:** Qinghong Li, James A. Holzwarth, Bethany Smith, Sonia Karaz, Mathieu Membrez, Vincenzo Sorrentino, Stacie Summers, Julie Spears, Eugenia Migliavacca

**Affiliations:** 1 Nestlé Purina Research, St. Louis, MO 63102, USA; 2 Nestlé Research, Lausanne, Switzerland; 3 Nestlé Purina Research, St. Joseph, MO 64503, USA; 4 Oregon State University, Corvallis, OR 97331, USA

**Keywords:** trimethylamine N-oxide, indoxyl sulfate, OAT1, OATP4C1, ABCC2

## Abstract

Aging leads to nephron senescence and chronic kidney disease (CKD). In cats, indoxyl sulfate (IxS) has been previously quantified and associated with CKD, and little is known about tubular transporters. Two cohorts of cats aged 6 to 21 years were enrolled. Cohort 1 included 41 colony cats with 28 control and 13 CKD cats. Cohort 2 had 30 privately-owned cats with 10 control and 20 CKD cats. In cohort 1, serum concentrations of IxS, trimethylamine N-oxide (TMAO), *p*-cresol sulfate (PCS), and phenyl sulfate were higher in CKD vs. control cats (all P<0.05). This observation was independently validated in cohort 2. Renal cortical and medullar tissues were collected from a third cohort of cats euthanized for humane reasons unrelated to the study. We provided the evidence that renal tubular transporter genes, OAT1, OAT4, OATP4C1, and ABCC2, but not OAT3, were expressed in the kidneys of cats, and their expressions were downregulated in CKD (all FDR<0.1). Cats and humans share 90.9%, 77.8%, and 82.5% identities in OAT1, OATP4C1, and ABCC2 proteins, respectively. In healthy cats, circulating TMAO and IxS are significantly correlated with age. Our study reveals impaired uremic toxin secretion and tubular transporter expression in cats with CKD.

## INTRODUCTION

Chronic kidney disease (CKD) is a naturally occurring kidney disease common in both geriatric cats and older people. Despite differing etiology, both species share many pathophysiological similarities, including chronic tubulointerstitial inflammation and fibrosis [1–3]. Most feline CKD cases are idiopathic with unknown primary causes, while in a minority of cases a specific underlying cause such as renal amyloidosis or neoplasia can be identified [4]. CKD is associated with an accumulation of gut microflora-produced uremic toxins (UTs), including trimethylamine N-oxide (TMAO), indoxyl sulfate (IxS), *p*-cresyl sulfate (PCS), phenyl sulfate (PS), and indole-3-acetic acid (IAA) [5–9]. Gut dysbiosis and impaired renal and intestinal barrier functions are thought to contribute to the plasma elevation of these UTs [5, 7, 10]. Previous studies have demonstrated that the concentration of circulating IxS was significantly higher in cats with CKD compared with healthy controls, and in cats with progressive CKD compared with non-progressive CKD [11–15]. In a recently untargeted metabolomic study, plasma TMAO level was increased in CKD cats compared to control cats, but protein bound uremic toxins (PBUTs), such as IxS, PCS, PS, and IAA, were not different between groups [16]. Indole-3-propionic acid (IPA), another indolic microbial metabolite, is thought to have many health benefits. But its involvement in CKD is not yet clear.

There are three classes of UTs as proposed by the European Uremic Toxin Work Group: small water-soluble compounds less than 500 Daltons, middle molecules 500-60,000 Daltons, and PBUTs, such as IxS, PCS, PS, and IAA, which exhibit high affinity for albumin [17, 18]. These UTs accumulate in the plasma when the kidneys are failing [19, 20]. TMAO, one of the small water-soluble uremic solutes, is synthesized from dietary carnitine, betaine and choline by gut bacteria and contributes to the development and mortality risk of cardiovascular disease and CKD in people [21–23]. While some small water-soluble uremic solutes can be removed by glomerular filtration, urinary excretion of PBUTs is mediated by active tubular secretion and with a range of transporters at the renal proximal tubules [24–26]. In humans and rodents, members of the organic anion transporter (OAT) and organic anion transporting peptide (OATP) families are uptake or influx transporters localized in the basolateral membrane of the proximal tubule, while members of the ATP binding cassette subfamily C (ABCC) are the main efflux transporters at the apical surface of the proximal tubule [27]. These transporters work in concert to move small organic anionic drugs, toxins, and other endogenous metabolites from the blood to the urinary lumen for excretions [28–31]. To date, no transporters have been identified in cats and how PBUTs are metabolized and handled in the feline kidneys is unclear.

In cats, multiple studies have highlighted age as one of the key risk factors for the development and progression of CKD [32–35]. CKD is most common in cats aged 12 years or older [4, 32]. We performed targeted serum metabolomics and renal tissue gene expressions to understand how the kidneys metabolize and eliminate UTs, including PBUTs, in cats. We tested the following hypotheses: (1) Serum concentrations of gut microflora-derived UTs are increased in CKD; (2) Urine UT levels are decreased in CKD; (3) Healthy cats aged 12 years or older have increased levels of UTs in their blood compared to younger ones. Furthermore, we explored the role of renal tubular transporters to gain insights on the mechanism of UT metabolism and handling in cats with CKD.

## RESULTS

The physical and clinical characteristics of cats are described in Table 1. All cats in cohort 1 were domestic short-haired. The top two breeds in cohort 2 were domestic short-haired (23/30) and domestic long-haired (3/30). In cohort 1, the control cats had a greater mean body weight than CKD cats, while no age difference was found between groups (P=0.005, P=0.15 respectively). In cohort 2, the control cats were younger, and body weight was not different between groups (P=0.001, P=0.09 respectively).

**Table 1:** Physical and clinical descriptions of the cats.

	**Cohort 1**				**Cohort 2**			
	**Control**	**CKD 2**	**P-value**		**Control**	**CKD 2**	**CKD 3**	**P-value**
Sample size	28	13	n/a		10	10	10	n/a
Age (years)	11.8±0.5	13.5±1.0	0.15		12.8±0.7^a^	17.7±0.6^b^	16.2±1.1^b^	0.001
Sex (F/M)	12/16	5/8	1.0		6/4	4/6	4/6	0.72
Weight (kg)	5.03±0.14	4.06±0.27	0.005		4.09±0.26	3.38±0.15	3.84±0.23	0.09
Breed								
DSH	28	13	n/a		10	6	7	n/a
DLH	0	0	n/a		0	2	1	n/a
Birman	0	0	n/a		0	1	0	n/a
Devon Rex	0	0	n/a		0	1	0	n/a
BSH	0	0	n/a		0	0	1	n/a
Tiffany	0	0	n/a		0	0	1	n/a
Clinical Measurement								
Serum creatinine	1.35±0.04	2.15±0.12	<0.001		1.28±0.05^a^	1.93±0.08^b^	3.56±0.23^c^	<0.001
Serum SDMA	11.8±0.4	20.9±1.5	<0.001		10.8±0.7^a^	17.9±1.5^b^	21.3±0.9^b^	<0.001
USG*	1.044±0	1.017±0	<0.001		1.046±0^a^	1.018±0^b^	1.015±0^b^	<0.001
UPC*	0.179±0.02	0.401±0.08	0.018		0.152±0.02	0.36±0.1	0.482±0.33	0.51
Proteinuria (N/Y/B)	19/7/1	4/3/6	0.003		8/2/0	5/2/3	7/2/1	0.56

CKD 2, stage 2 chronic kidney disease; DSH, domestic short-haired; DLH, domestic long-haired; BSH, British short-haired; SDMA, symmetric dimethylarginine; UPC, urine protein to creatinine ratio; USG, urine specific gravity. P-values were obtained from student’s t test (cohort 1) and ANOVA test (cohort 2). Different superscripted letters in cohort 2 denote significant statistical difference between groups by Tukey’s multiple comparisons while the same letters indicate no significant difference. Continuous variables are reported as mean±SEM. Feline proteinuria according to the IRIS guidelines: non-proteinuric (N) (UPC<0.2); borderline proteinuric (B) (UPC [0.2, 0.4]); proteinuric (Y) (UPC>0.4). Units for serum creatinine and SDMA are mg/dL and μg/dL, respectively.

*urine sample from a control cat in cohort 1 was not collected.

The two cohorts differed significantly in urine protein:creatinine (UPC) ratio (Table 1). Proteinuria was determined using the same parameter for both healthy and CKD cats according to the International Renal Interest Society (IRIS) guidelines. In cohort 1, UPC was higher in cats with CKD compared to healthy control cats (P=0.018): 19/27 and 7/27 healthy cats were non-proteinuric (UPC<0.2) and borderline proteinuric (UPC≥0.2 and ≤0.4), respectively, while 6/13 CKD cats were proteinuric (UPC>0.4). In cohort 2, UPC was not different among groups (P=0.51): 8/10 healthy control cats were non-proteinuric, and 5/10 and 7/10 cats with CKD stage 2 and stage 3, respectively, were non-proteinuric.

### Serum metabolites

**Figure 1:**
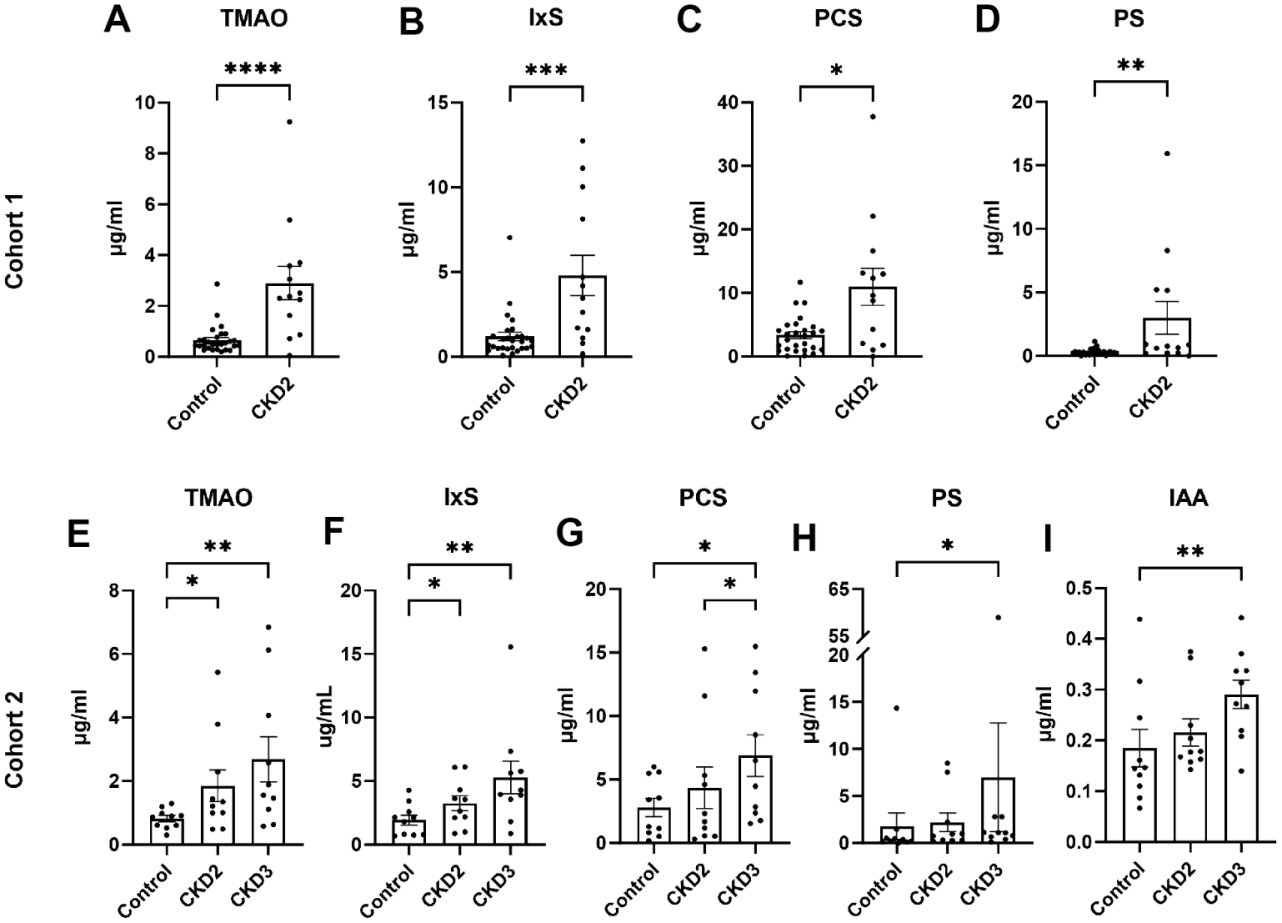
**Serum concentrations of uremic toxins.** The concentrations of uremic toxins were quantified in cohort 1 (**A**–**D**) and cohort 2 (**E**–**I**). Asterisks (*) indicate statistical significance from (**A**–**D**) Mann-Whitney test and (**E**–**M**) Dunn’s multiple comparisons test: *P<0.05, ** P<0.01, *** P<0.001, **** P<0.0001. The box and bars represent mean and SEM. TMAO, trimethylamine N-oxide; IxS, indoxyl sulfate; PCS, *p*-cresol sulfate; PS, phenyl sulfate; IAA, indole-3-acetic acid; CKD2, CKD stage 2; CKD3, CKD stage 3.

In cohort 1, the concentrations of TMAO, IxS, PCS, and PS increased more than threefold in cats with CKD stage 2 compared with healthy control cats (P<0.05 in all cases, Figure 1A–1D and Supplementary Table 1).

In cohort 2, the concentrations of TMAO and IxS increased more than 1.6-fold in cats with CKD stages 2 and 3 when compared to control cats (Figure 1E, 1F and Supplementary Table 2). The concentrations of PCS, PS, and IAA were increased in CKD stage 3 cats compared to control cats (Figure 1G–1I).

No difference was observed in indole-3-propionic acid (IPA) between groups.

### Urine metabolites

**Figure 2:**
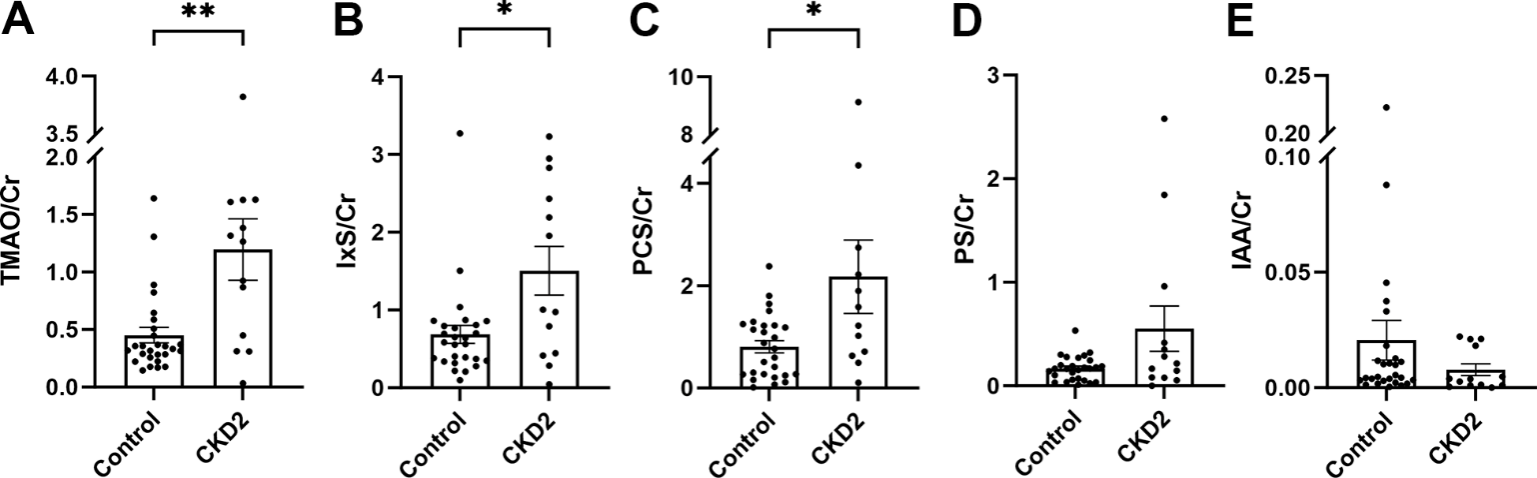
**Urine levels of uremic toxins in cohort 1.** The levels of urinary uremic toxins were normalized by creatinine (**A**–**E**). The box and bars represent mean and SEM. TMAO, trimethylamine N-oxide; IxS, indoxyl sulfate; PCS, *p*-cresol sulfate; PS, phenyl sulfate; IAA, indole-3-acetic acid; Cr, creatinine; CKD2, CKD stage 2. Asterisks (*) denote P-values from the Mann-Whitney test, *P<0.05, **P<0.01.

In cohort 1, the levels of TMAO, IxS, and PCS, indexed by creatinine, were increased in cats with CKD stage 2 compared to control cats (Figure 2A–2C and Supplementary Table 3). No difference between groups was observed in PS or IAA (Figure 2D, 2E). The IPA level was under the detection limit.

No difference between groups was found in cohort 2 (Supplementary Table 4).

### Association between age and uremic toxins in healthy cats

**Figure 3:**
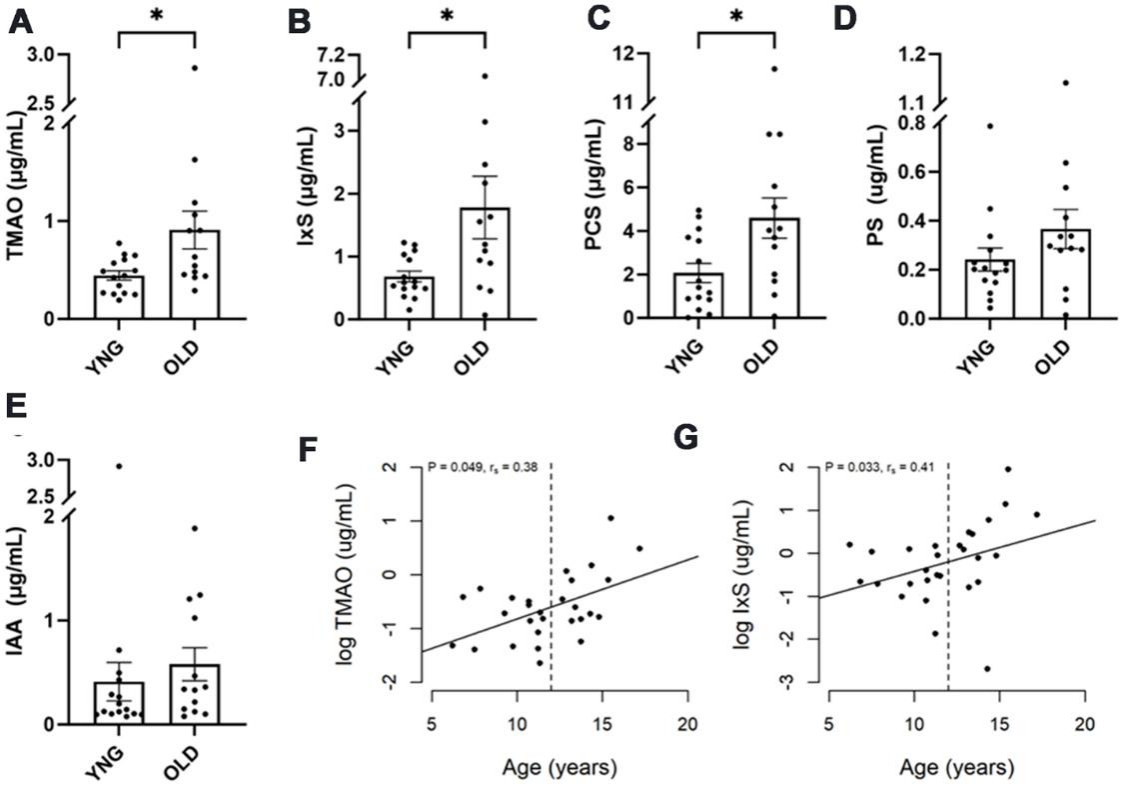
**Uremic toxins in healthy senior cats.** Healthy cats in cohort 1 were divided into two groups: those less than 12 years of age (YNG), and those aged 12 years or older (OLD). (**A**–**E**) The box and bars indicate means and SEM. P-values from the Mann-Whitney test are presented as asterisks: *P < 0.05. (**F**, **G**) Spearman’s correlation analysis between cat’s age and log concentration of TMAO and IxS, respectively. P-value and correlation coefficient are indicated in the plot.

The 28 healthy control cats from cohort 1 were divided into two groups by the age of 12 years, resulting 13 cats in the older group (OLD) and 15 cats in the younger group (YNG). Serum concentrations of TMAO, IxS, and PCS were more than doubled in OLD compared to YNG (P = 0.036, 0.049, and 0.033, respectively, Figure 3A–3C and Supplementary Table 5). No change between groups was observed in PS, IAA, or IPA (Figure 3D, 3E).

Age was positively associated with the levels of TMAO and IxS in the healthy cats in cohort 1 (P=0.048, 0.033; *r_s_*=0.38, 0.41, respectively, Figure 3F, 3G).

In cohort 2, where 10 healthy control cats were equally split between YNG and OLD, no difference in UTs was found between the age groups.

### Analysis of confounding effects

Because CKD is most common in geriatric cats older than 12 years, a bootstrap resampling simulation was performed to assess age confounding effect on CKD. Out of 1000 bootstrap-generated sub-data sets, 977 showed no difference in age or sex between control and CKD groups (Supplementary Figure 1A and Supplementary Table 6). In all cases, body weight remained different between groups. Serum concentrations of TMAO, IxS, PCS, and PS were significantly different in all 977 sub-data sets (FDR<0.05 in all cases, Supplementary Figure 1B–1E and Supplementary Table 6).

### Renal tissue RNA-seq and RT-qPCR experiments

In cortex, the median expressions of OAT1 and OAT4 were 586 counts per million (CPM) and 804 CPM, respectively, while the median expressions of OATP4C1 and ABCC2 were 33 CPM and 7 CPM, respectively (Supplementary Table 8A). In medulla, the median expressions of OAT1 and OAT4 were 16 and 267 CPM, respectively, while the median expressions of OATP4C1 and ABCC2 were 13 and 7 CPM, respectively (Supplementary Table 9A).

**Figure 4:**
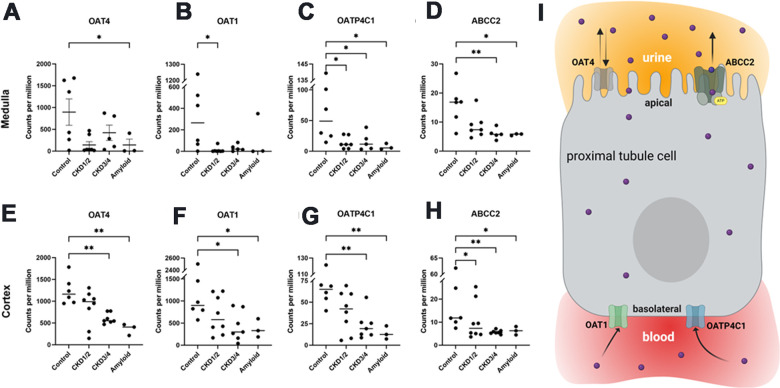
**Organic anionic transporter RNA-seq gene expressions.** Medullar (**A**–**D**) and cortical (**E**–**H**) tissue expressions of OAT4, OAT1, OAPT4C1, and ABCC2, respectively. Illustration of transporters in the proximal tubule cell (**I**). OAT1 and OATP4C1 are the uptake transporters on the basolateral surface, while ABCC2 is the efflux transporter localized at the apical surface. OAT4 is thought to play a role in both reabsorption and secretion from the apical side. All genes were differentially expressed between control and across CKD groups in both cortex and medullar (FDR<0.1 in all cases). Bars indicate means. Asterisks indicate Dunn’s multiple comparisons tests: *P<0.05, **P<0.01. OAT1/4, organic anion transporter family member 1/4; OATP4C1, organic anion transporting peptide family member 4C1; ABCC2, ATP-binding cassette subfamily C member 2. CKD1/2: CKD stages 1 and 2; CKD3/4: CKD stages 3 and 4; Amyloid: CKD by amyloidosis. Panel I created with BioRender.com.

In cortex, the expressions of OAT4, OAT1, OATP4C1, and ABCC2 were lower in CKD3/4 and Amyloid groups compared to control group (FDR<0.1, Figure 4E–4H and Supplementary Table 8B, 8C).

In medulla, OAT1and OATP4C1 expressions were lower in CKD1/2 group compared to control group ABCC2 expression was lower in CKD3/4 and Amyloid groups compared to control group (FDR<0.1, Figure 4A–4C, Supplementary Table 9B, 9C).

The six control cats were divided into two groups, those older than 12 years (OLD, n=4) and those younger than 12 (YNG, n=2). The mean expressions of OAT1 and OAT4C1, two influx transporters at the basolateral surface (Figure 4I), showed a trend of downregulations in the OLD group compared with YNG (Supplementary Tables 8D, 9D).

RT-qPCR was performed on three genes: OAT1, OAT3, and OATP4C1. The observations on OAT1 and OATP4C1 in RNA-seq were supported by the RT-qPCR assay (Supplementary Figure 2).

Surprisingly, the OAT3 expression in RNA-seq was very low in both tissues, such that it did not reach the minimum expression threshold. The observation was confirmed by the RT-qPCR results: medullar OAT3 was undetectable, while the cycle threshold value for cortical OAT3 was too high to merit further analysis.

### Protein sequence alignments

**Figure 5:**
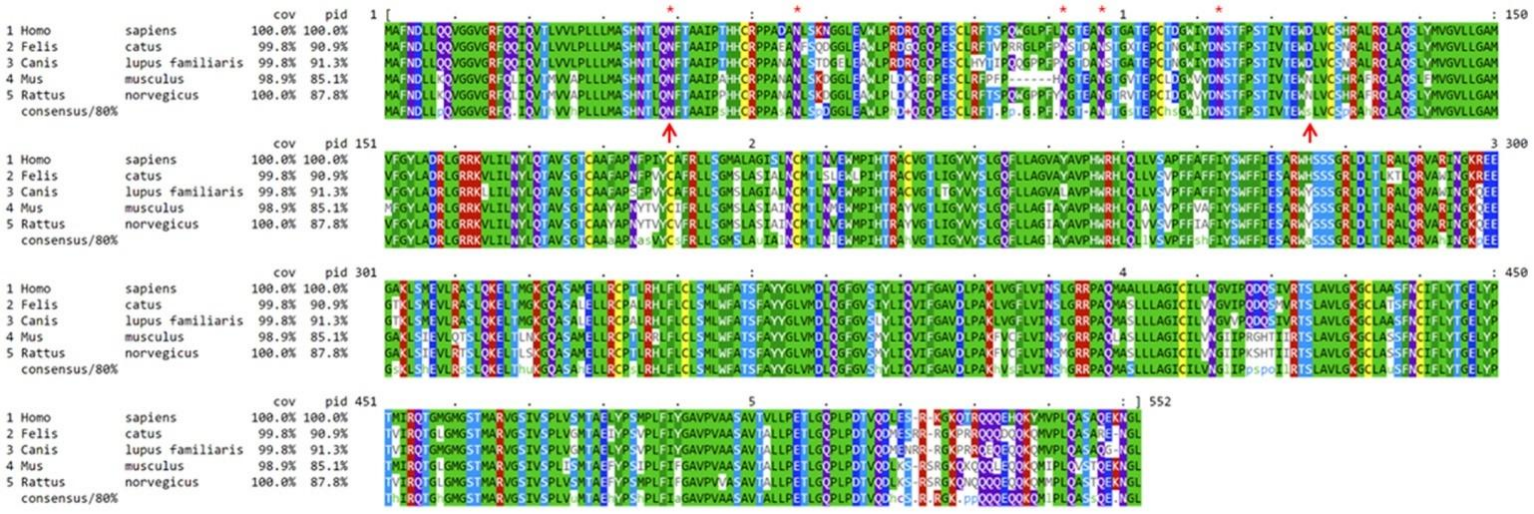
**Alignment on mammalian OAT1 protein sequences.** Mouse, rat, dog, and cat OAT1 protein sequences are compared to their human orthologous sequence. Dogs and cats share 91.3% and 90.9% OAT1 protein sequence identities with humans, respectively. “Cov” indicates the percentage of amino acids covered in the alignment, and “pid” indicates protein sequence identity compared to humans, consensus denotes consensus out of 80% of the sequences from the group. Red asterisks indicate the five glycosylation sites on the first extracellular loop, which is delineated by the two upward red arrows in positions 39 and 125, respectively. Humans share the same hydrophilic aspartate (D) with cats and dogs (marked by the 2^nd^ red arrow), but not rodents. Dog and cat’s OAT1 proteins are computationally predicted.

The OAT1, OATP4C1, and ABCC2 proteins are evolutionarily conserved among humans, mice, rats, dogs, and cats. The computationally annotated feline OAT1, OAT4C1, and ABCC2 proteins share 90.9%, 78.9%, and 82.5% sequence identities at the amino acid level with their human orthologs, respectively (Figure 5 and Supplementary Figures 3, 4). Remarkably, canine OAT1 protein is 91.3% identical to the human ortholog (Figure 5). At the phylogenetic level, the feline proteins are more similar to the canine orthologs when compared to humans and rodents (Supplementary Figure 5).

## DISCUSSION

A scoring system has been developed to categorize the strength of cellular toxicity of UTs [36]. Those with high scores included small water-soluble TMAO and protein-bound IxS, PCS, PS, and IAA. These UTs have been implicated as a risk factor for kidney injury and mortality. Our results supported the first hypothesis that serum concentrations of several major UTs increased in cats with CKD. Importantly, our findings in colony cats were independently validated in the privately-owned cats, and are consistent with those from people and animal models with CKD [37]. Nealson et al. showed in a recent untargeted metabolomic study that serum concentration of TMAO increased 20% in CKD cats compared to healthy control cats [16], whereas the targeted approach showed a more than twofold increase in this study. In addition, PBUTs, including IxS that has been previously associated with feline CKD [11–15], were not different between groups [16]. These discrepancies are likely due to the difference between untargeted and targeted metabolomic approaches. In general, a targeted approach is more sensitive, precise, and specific than an untargeted approach, and is more likely to detect a statistically significant perturbation [38, 39].

Contrary to our second hypothesis, urine concentrations of several UTs, indexed to creatinine, were higher in cats with CKD stage 2 compared to the control in cohort 1, but no difference was found between groups in cohort 2. In a recent study in which human patients with CKD were compared with age, sex, and race-matched controls [37], although serum concentrations of TMAO, IxS, and PCS were higher in the CKD group compared with control group, urinary uremic toxins-to-creatinine ratios were not different between groups. In another study, serum TMAO was higher in CKD patients compared to controls, yet no difference in urinary TMAO/creatinine ratio was observed between groups [40]. These observations are consistent with ours in cohort 2, but not cohort 1. Although it is a common practice to normalize urine solute concentrations by urine creatinine, assuming creatinine is constant over time and within an individual, multiple factors can influence urine concentration and flow. Cats with CKD often have an increase in thirst (polydipsia) and frequent urination (polyuria), which may have an impact on urine solute concentrations. Although quite challenging, a timed urine collection over a 24-period would provide more accurate measurements of metabolite concentrations than single spot sampling [41, 42]. More studies and a better standardized sampling approach are necessary to understand these discrepancies.

CKD is an age-dependent decline of renal functions, and most common in cats over 12 years of age [4, 32, 43]. As a result, the remaining nephrons must work at a higher capacity to maintain a normal total glomerular filtration rate (GFR) [44]. It has been proposed that the development of CKD could be viewed as a lengthy and integral part of the aging process in domestic cats [45]. If reduced renal clearance is one of the key contributors to the retention of blood UTs, then the levels of circulating UTs should be higher in senior vs. young cats. Thus, we tested the third hypothesis that the concentrations of serum UTs are increased with age. In cohort 1, where there were 28 healthy control cats were roughly equally divided by the age of 12 years, our data showed that serum levels of TMAO, IxS, and PCS were increased in cats older than 12 years of age when compared to the younger cats. In addition, serum TMAO and IxS were positively correlated with age. We repeated the same analysis in cohort 2, where 10 healthy cats were equally split, but did not find any difference between groups. This is likely due to the difference in sample size: cohort 1 had nearly 3 times as many healthy control cats as cohort 2. In addition, we analyzed the renal tissue RNA-seq data from the six control cats. Although the sample size was small, our analysis suggested that there was a trend of downregulations in the two influx transporters, OAT1 and OATP4C1, localized in the basolateral membrane of the proximal tubule in cats 12 years or older compared with younger cats. Thus, it is possible that the changes in the transporter gene expressions may also contribute to the changes in uremic toxins in older cats.

No difference was observed in IPA. In cohort 2, serum concentration of IAA was increased in CKD stage 3, but not stage 2, when compared to the control group. Both IAA and IPA belong to the family of the gut microbe-produced indolic metabolites. IAA is a UT and its serum concentration was increased in human patients with CKD [46]. The level of IAA was positively correlated with markers of inflammation and oxidative stress [47]. Serum IAA concentration was considered as an independent predictor of mortality and cardiovascular events in people with CKD [47]. In contrast, IPA is a beneficial indole derivative which inhibits atherosclerosis in humans and promotes nerve regeneration and repair in mice, but its involvement in CKD is not yet clear [48, 49]. In cats, serum and urine concentrations of IAA and IPA were considerably lower compared to other metabolites such that urine concentrations of IPA were below our detection limit.

To address the potential age confounding effect on circulating UTs in CKD cats, we performed 1000 iterations of bootstrap resampling experiments in cohort 1. In all bootstrapped sub-data sets (977/1000) where age was not different between groups, the concentrations of TMAO, IxS, PCS, and PS were significantly different between CKD and control groups. Our analysis demonstrated that the observed differences in UTs between the control and CKD groups were independent of age. Cats with CKD usually experience thin body condition and weight loss, which can be detected before diagnosis and accelerates after diagnosis [48]. Body weight was not different between groups in cohort 2 (P=0.09), and yet the serum concentrations of multiple UTs were significantly different between groups, suggesting the observed difference in UTs is not likely due to body weight.

The role of the renal organic anion transporters OAT1 (also known as SLC22A6 or NKT) and OAT3 (aka SLC22A8) are still poorly understood. In humans and rodents, both OAT1 and OAT3 are predominantly and strongly expressed in the basolateral membrane of the proximal tubular cells, and share a high protein sequence identity with each other [28, 31, 49]. In physiological conditions, the metabolism and excretion of organic anionic solutes, including some drugs and PBUTs, are handled by the concerted effort of several classes of transporters to enable the movement of these compounds from the blood to the urinary lumen at the proximal tubules (illustrated in Figure 4I) [31, 50]. These transporters include members of organic anion transporter (OAT), organic anion transporting peptide (OATP), and ATP-binding cassette subfamily C (ABCC) transporter families [50]. In the proximal tubule cells, OAT1/3, and OATP4C1 (aka SLCO4C1) are uptake or “influx” transporters localized at the basolateral surface to move drugs or toxins from the blood, while the ATP-binding cassette subfamily C member 2 (ABCC2 or MRP2) and member 4 (ABCC4 or MRP4) are the main “efflux” transporters localized at the apical surface to move those solutes to the urinary lumen for excretion [27]. Currently, our knowledge on OAT1-3 mostly came from the studies using cell culture or rodent models, such as knock-out mice and nephrectomy rats [29, 51–53]. In *Oat1* and *Oat3* knock-out mice, plasma levels of many UTs were increased [52]. The expression of OATP4C1, which is a member of OATP family localized at the basolateral membrane of the proximal tubule cell [30], was reduced in human and rodent models of renal failure [28, 54]. Overexpression of human OATP4C1 reduced UT accumulation in rats [55].

In cats, data on renal transporters were lacking. For the first time, we provided evidence that OAT1, OAT4, OATP4C1, and ABCC2, but not OAT3, are expressed in the kidneys of cats, and their expressions were downregulated in CKD. Amyloid protein depositions in the kidneys can progressively impair renal structure and function, leading to severe proteinuria and rapid progression to end-stage in both human and feline patients [54, 56]. Our data showed that cats with CKD secondary to amyloidosis had impaired renal tissue transporter gene expressions comparable to those with advanced stage of CKD.

We further demonstrated that OAT1, OAT4C1, and ABCC2 proteins are highly conserved across mammalian species. Given that OAT3 was expressed almost exclusively and abundantly in the kidneys of humans and rodents, it was surprising that OAT3 was expressed at such a low level, if at all, in the kidneys of cats. It will be interesting to know whether OAT3 protein is expressed in cats. Additionally, renal tissue ABCC4 was not differentially expressed between groups in cats. Taken together, our study suggests that although many of the transporters are highly conserved across mammalian species, the mechanism and pathway in which the kidneys metabolize and handle organic anionic metabolites may have diverged during evolution.

There are some important differences between the two cohorts of cats. The cats in cohort 1 all lived in the same environment in a research colony where many variables were controlled and they received routine veterinary care, while cats in cohort 2 lived in different homes thereby increasing environmental variability. In cohort 1, cats with CKD had a significant increase in UPC compared to the healthy control cats, while the vast majority of CKD cats in cohort 2 were either non-proteinuric or borderline proteinuric. This large variability in proteinuria in cats that lived in different homes was likely compounded by spot urine sampling. Fasting blood samples were collected from the colony cats, but not for cats living in private homes. Diet was not controlled in this study. While the majority of cats with CKD were on a veterinary diet with a reduced amount of high-quality protein to support kidney function, a small percentage of CKD cats were on different adult maintenance diets. Another limitation is that the cats in the gene expression study were not the same cats as in the targeted uremic toxin study. Therefore, we were not able to determine any direct association between these organic anionic transporters and uremic toxin excretions. Unlike rodent models of renal disease, feline CKD is a naturally occurring renal disease, which share many pathophysiological similarities to human CKD. Learning from cats on this age-associated health condition could be applied to improve and maintain kidney health in both cats and humans.

## MATERIALS AND METHODS

### Animals and study design

Serum and urine samples were collected from two cohorts of cats. All cats in cohort 1 were colony cats in North America, including 28 healthy control cats and 13 cats with CKD IRIS stage 2 (Table 1). All cats in cohort 2 were privately-owned cats in Europe, equally split between 3 groups of healthy control, CKD IRIS stage 2 and stage 3.

Renal tissues were collected from a third cohort of cats in North America that were euthanized for humane reasons unrelated to the study. The cortex tissues were collected from 24 cats, including 6 healthy control cats, 8 cats with CKD stages 1 and 2 (CKD1/2), 7 cats with CKD stages 3 and 4 (CKD3/4), and 3 cats with CKD by amyloidosis (Amyloid) cats. The medulla tissues were collected from 6 control cats, 7 cats with CKD1/2, 5 cats with CKD3/4, and 3 Amyloid cats. Amyloid cats were diagnosed using renal pathology data.

The study was approved by the Institutional Animal Care and Use Committee of Nestlé Purina PetCare Company. For each privately-owned cat, a signed owner informed consent was obtained. Cats with major systemic diseases such as cancer, heart failure, or diabetes mellitus were excluded from the study.

### Diagnosis and staging of CKD

To be eligible, all cats underwent a thorough evaluation including a review of medical history, a complete physical examination, a complete blood count and serum biochemistry panel which included creatinine, symmetric dimethylarginine (SDMA) and total thyroxine, indirect blood pressure measurement, urinalysis including urine specific gravity (USG) as well as urine protein to creatinine ratio (UPC). A cat was considered to have CKD if USG <1.035, and either serum creatinine >1.6 mg/dL or serum SDMA >14 μg/dL on at least two different assessments and the history and physical examination findings supported a diagnosis of CKD. After a diagnosis of CKD was made, staging of CKD followed the IRIS guidelines and recommendations. Cats with amyloidosis were diagnosed using additional renal pathology data. A cat was considered healthy if there was no history or physical examination finding compatible with kidney or urinary tract disease or other significant medical condition and met all the following criteria at the time of enrollment: USG >1.035, serum SDMA <14 μg/dL, serum creatinine <1.6 mg/dL, and systolic blood pressure <160 mmHg. A cat with prior history of azotemia but was resolved at the time of sampling was not considered as control.

For cats in cohort 3, CKD diagnosis and staging were performed based on renal pathology report, health history, and clinical measurements at the time of euthanasia. Cats with stage 1 CKD had renal pathologies consistent with chronic tubulointerstitial nephritis, blood SDMA <18 ug/dL and creatinine <1.6 mg/dL.

The CKD in all three cohorts was naturally occurring.

### Sample collections

Fasting blood and urine samples were collected from cats in cohort 1. Cats in cohort 2 were not fasted at the time of sample collections. Urine samples were collected via cystocentesis. Samples were collected between 2017-2022, and were stored in -80° C until use.

### Targeted uremic toxin analysis

Total serum and urine concentrations of IxS, TMAO, PCS, PS, IAA, and indole-3-propionic acid (IPA) were determined by HPLC-MS/MS as previously described and in the Supplementary Method [11, 57]. Urine metabolite concentrations were normalized by urine creatinine concentration.

### Statistical analysis of metabolites

Data normality was evaluated using Shapiro-Wilk test. For non-normal variables, the Mann-Whitney test was used to compare the means of two groups, or the Kruskal-Wallis test followed by Dunn’s multiple comparisons test were used to compare three or more groups. For normal variables, Student’s t test was used to compare two groups, otherwise, ANOVA test followed by Tukey’s multiple comparisons test were used. The χ^2^ or Fischer’s exact test was performed to test the null hypothesis that categorical variables were independent.

The healthy control cats from cohort 1 were divided into two groups, those younger than 12 years of age (YNG) and those aged 12 or older (OLD). The Mann-Whitney test was used to compare the means between the two age groups. Spearman’s correlation analysis was performed to determine the relationship between age and serum UT concentrations.

Spearman’s rank correlation test was also applied to test the relationship of metabolites with each other and their relationship with clinical variables, including serum creatinine, SDMA, UPC, and USG. P-values were adjusted for multiple testing as false discovery rate (FDR) using the Benjamini-Hochberg method.

### Evaluation of confounding effects

To address the potential confounding effects from age or body weight on circulating UTs, 1000 iterations of bootstrapping without replacement experiments were performed on the 28 control samples in cohort 1. In each iteration, 20 samples were randomly chosen and combined with the 13 CKD samples to generate a new sub-data set. On each sub-data set, Mann-Whitney test was performed to determine the mean difference of serum UT concentrations were different between groups. The P-values were adjusted for multiple testing using FDR.

### Renal tissue RNA extraction, RNA-seq, and RT-qPCR

Twenty-four cortical and 21 medullar tissue samples were collected from age and sex-matched CKD and non-CKD cats (Supplementary Table 7). Renal tissue total RNA was extracted using the Agencourt RNAdvance Tissue Kit (Beckman Coulter, USA). The RNA was quantified using Quant It Ribogreen assay (Life Technologies, USA), and its quality was checked on a Fragment Analyzer (Agilent, USA). The RNA samples with low RNA quality numbers (RQNs) were excluded from further analysis. The cDNA library was constructed by following Illumina Stranded mRNA Library Prep protocol workflow. The library size was controlled with the High Sensitivity NGS Fragment Analysis kit on a Fragment Analyzer. The libraries were quantified with Qubit dsDNA HS Assay Kits (Thermo Fisher Scientific, USA). Samples were pooled equimolar. Sequencing was performed on six flow cells on Illumina NextSeq 2000 with V3 chemistry PE 150 cycles to achieve an average of 125 million reads per sample.

### Analysis of RNA-seq and RT-qPCR gene expression data

Sequencing raw data were demultiplexed and transformed into fastq files using casava v1.8.2. The fastq files were then aligned against the domestic cat reference genomes Felis_catus_9.0 using RNAstar v2.5.3. The counts per gene were generated using htseq_count v2.16.2. Genes with less than 2 counts per million in at least 5 samples were discarded. The remaining sequencing data were normalized by the trimmed mean of M-values (TMM) method as implemented in the function *calcNormFactors* in edgeR [58]. Differentially expressed genes were defined by fitting a quasi-likelihood negative binomial generalized log-linear model to count data using the *glmQLFTest* function also implemented in the R package “edgeR”.

The qPCR primers, and protocol are described in the Supplementary Methods. PCR data were analyzed using the Dunn’s multiple comparisons test.

### Comparative analysis of transporter sequences

The protein sequences for OAT1, OATP4C1, and ABCC2 were obtained from NCBI by searching for the human sequence and using the orthology function to extend to other mammalian species, including *Felis catus, Canis lupus familiaris, Mus musculus,* and *Rattus norvegicus*. Alignment was performed using COBALT, and visualization of the alignment using mview (version 1.64) [59, 60]. The phylogenetic tree was created using FastTree in several stages [61]. First, it uses Heuristic neighbor-joining, which is followed by a reduction in the length of the tree using a mix of nearest-neighbor interchanges and subtree-prune-regraft moves. Thirdly, it maximizes the tree’s likelihood and finally takes into consideration local support values using the Shimodaira-Hasegawa test.

## Supplementary Material

Supplementary Methods

Supplementary Figures

Supplementary Table 1

Supplementary Table 2

Supplementary Table 3

Supplementary Table 4

Supplementary Table 5

Supplementary Table 6

Supplementary Table 7

Supplementary Table 8

Supplementary Table 9
