# Supplementary material for: Impaired renal transporter gene expression and uremic toxin excretion as aging hallmarks in cats with naturally occurring chronic kidney disease
Source: Aging (Albany NY). 2024 Dec 20;16(22):13588–607. doi: 10.18632/aging.206176 (PMC11723653; doi:10.18632/aging.206176)
Supplement: Supplementary Methods [file aging-16-206176-s001.pdf]

SUPPLEMENTARY METHODS

Targeted metabolomics assay by HPLC-MS/MS

Forty microliters (40 uL) of serum were mixed with 10 µL of internal standard and 200 µL of cold methanol. The mixture was vortexed for 5 seconds and then incubated at -20° C overnight, followed by centrifugation at 15000 g and 4° C for 15 minutes. One-hundred 100 uL supernatant was recovered and mixed with 900 µL of 50% methanol, which was stored at -20° C until analysis. Twenty microliters (20 uL) of urine sample were mixed with 80 µL of cold methanol, and then incubated at -20° C overnight, followed by centrifugation at 15000 g and 4° C for 15 min. The supernatant (10 µL) was recovered and then added with 10 µL of internal standard and 1 mL of 50% methanol, which were then stored at -20° C until analysis. The internal standard included 12.5 µg/mL of TMAO-D9, 250 µg/mL of PCS-D7, 12 µg/mL of IxS-D5, and 1 µg/mL of IAA-D5 dissolved in 50% methanol in water. Details of the assay can be found in the Supplemental Methods. Serum concentrations of metabolites are reported as µg/mL.

The authentic standards PCS was purchased from APExBio Technology, IS and IS-D5 from Cayman Chemical, TMAO, IAA and IPA from Sigma-Aldrich, TMAO-D9 from Santa Cruz Biotechnology, PCS-D7 from Cambridge Isotope, IAA-D5 from CDN Isotope, PS from TCI Chemicals. Uremic toxin internal standard mix contained 12.5 µg/mL of TMAO-D9, 250 µg/mL of pCS-D7, 12 µg/mL of IDS-D5, and 1 µg/mL of IAA-D5 dissolved in 50% methanol in water.

The serum sample (40 µL) was mixed with 10 µL of internal standard and 200 µL of cold methanol. The mixture was vortexed for 5 s and then incubated at -20° C overnight, followed by centrifugation at 15000 g and 4° C for 15 min. The supernatant (100 µL) was recovered and then added with 900 µL of 50% methanol, which were then stored at -20° C until analysis. A small aliquot of sample extract was pooled from each sample to generate a quality control (QC) sample.

UPLC-MS/MS was performed on a Waters Acquity UPLC coupled to a Waters Xevo TQ-S triple quadrupole mass spectrometer. Chromatographic separations were carried out on a Waters UPLC T3 stationary phase (2.1 x 50 mm, 1.8 µM) column. Mobile phases were water with 0.1% formic acid (A) and acetonitrile with 0.1% formic acid (B). The analytical gradient was as follows: time = 0 min, 1% B; time = 0.65 min, 1% B; time = 2.85 min, 99% B; time = 3.5 min, 99% B; time = 3.55 min, 1% B; time = 5 min, 1% B. Flow rate was 500 µL/min. Samples were held at 6° C in the autosampler, and the column was operated at 45° C. Injection volume was 2 µL. The capillary voltage of MS detector was set to 0.7 kV MS in both positive and negative mode during polarity switching. Inter-channel delay was set to 3 msec. Source temperature was 150° C and desolvation temperature 550° C. Desolvation gas flow was 1000 L/hr, cone gas flow (nitrogen) was 150 L/hr, and collision gas flow (argon) was 0.15 mL/min. Nebulizers pressure (nitrogen) was set to 7 Bar. Autodwell feature was set for the collection of 12 points-across-peak. Cone voltage and collision energy of each MRM was manually optimized.

Renal tissue gene expression assay by RT-qPCR

RNA was isolated from snap frozen kidney cortex and medulla. cDNA was reverse-transcribed from 250 ng of RNA using the High-Capacity cDNA Reverse Transcription Kit (Thermo Fisher Scientific) following manufacturer's instructions, and undiluted cDNAs were used to perform quantitative RT-PCR on a LightCycler® 480 (Roche) using SYBR Green PCR Master Mix (Roche). The pre-incubation phase was performed at 95° C for 7 min and cDNAs were then amplified over 45 cycles at 95° C for 15 s and 63° C for 1 min. To quantify mRNA expression, 2-ΔΔCt calculation was used to express results relative to HPRT and RPL17 expression as these reference genes were previously selected based on their stability across specific cat tissue (Penning et al; 2007). The following primer sequences were used:

HPRT
Forward: 5'- ACTGTAATGACCAGTCAACAGGGG-3'
Reverse: 5'- TGTATCCAACACTTCGAGGAGTCC-3'
RPL17
Forward: 5'- CTCTGGTCATTGAGCACATCC-3'
Reverse: 5'- TCAATGTGGCAGGGAGAGC-3'
OAT1
Forward: 5'- GGGTGATACCCCAGGATCAGT-3'
Reverse: 5'- CAGTTGAAGGAGGTAGCCAGG-3'
OAT3
Forward: 5'-AAACCATCTGGGCCAACACA
Reverse: 5'-GGTCTGCGAATCCGAGGG
OATP4C1
Forward: 5'-GGAGGAGTGAGTGGGAGAGT -3'
Reverse: 5'-GCCATTTACTACAATACCTTCTGC-3'
