# Supplementary material for: Impaired renal transporter gene expression and uremic toxin excretion as aging hallmarks in cats with naturally occurring chronic kidney disease
Source: Aging (Albany NY). 2024 Dec 20;16(22):13588–607. doi: 10.18632/aging.206176 (PMC11723653; doi:10.18632/aging.206176)
Supplement: Supplementary Figures [file aging-16-206176-s002.pdf]

SUPPLEMENTARY FIGURES

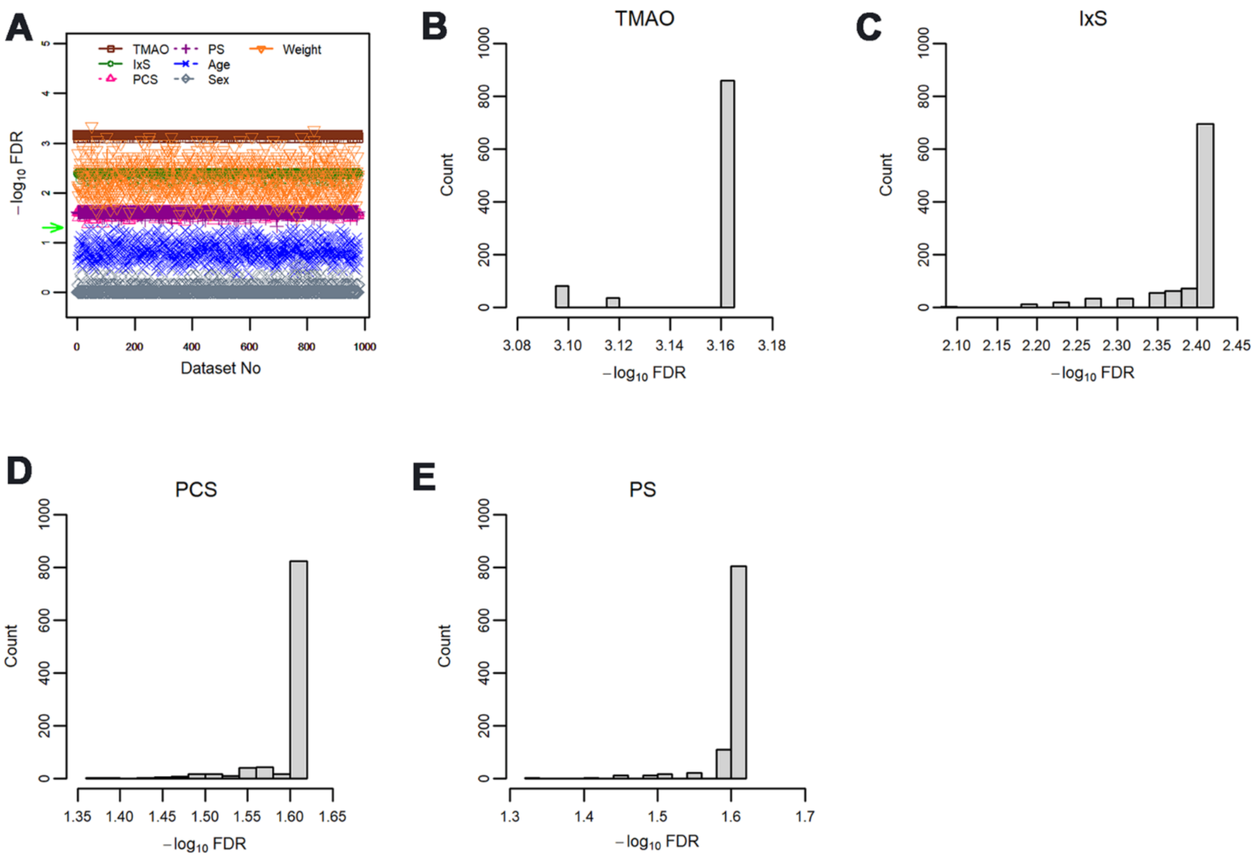

Supplementary Figure 1. Distribution of false discovery rate (FDR) from bootstrap resampling experiments. (**A**) Out of 1000 bootstrap resampling experiments, 977 sub-data sets showed no difference in age (blue) or sex (grey) between CKD and control groups. Body weight (orange) remained different between groups in all sub-data sets. The green arrow to the left denotes FDR=0.05 [$\log_{10}(0.05) = 1.3$]. (**B**–**E**) Histograms of $-\log_{10}$ (FDR) for TMAO, IxS, PCS, and PS, respectively.

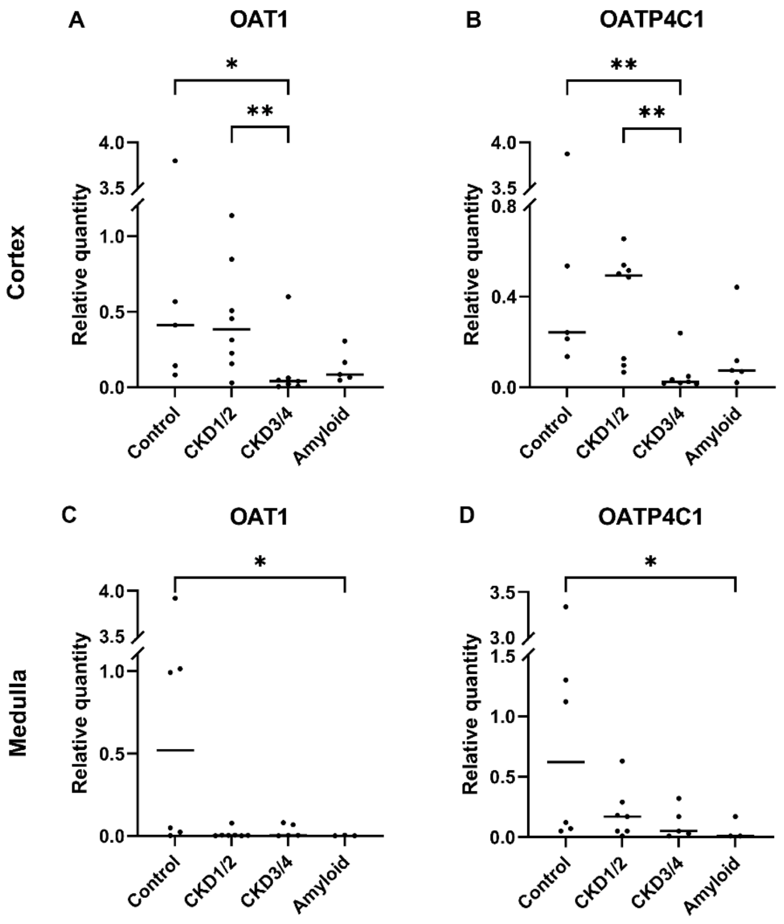

Supplementary Figure 2. Renal tissue gene expressions by RT-qPCR. Relative expressions of OAT1 and OATP4C1 in cortex (**A**, **B**) and medulla (**C**, **D**) (all FDR<0.1). CKD stages 1 and 2; CKD3/4: CKD stages 3 and 4; and Amyloid: CKD by amyloidosis. Asterisks indicate Dunn's multiple comparisons tests: *P<0.05, **P<0.01. OAT3 was not detected in the medullar tissue, and its expression level in the cortex was very low, if any (high CT value).

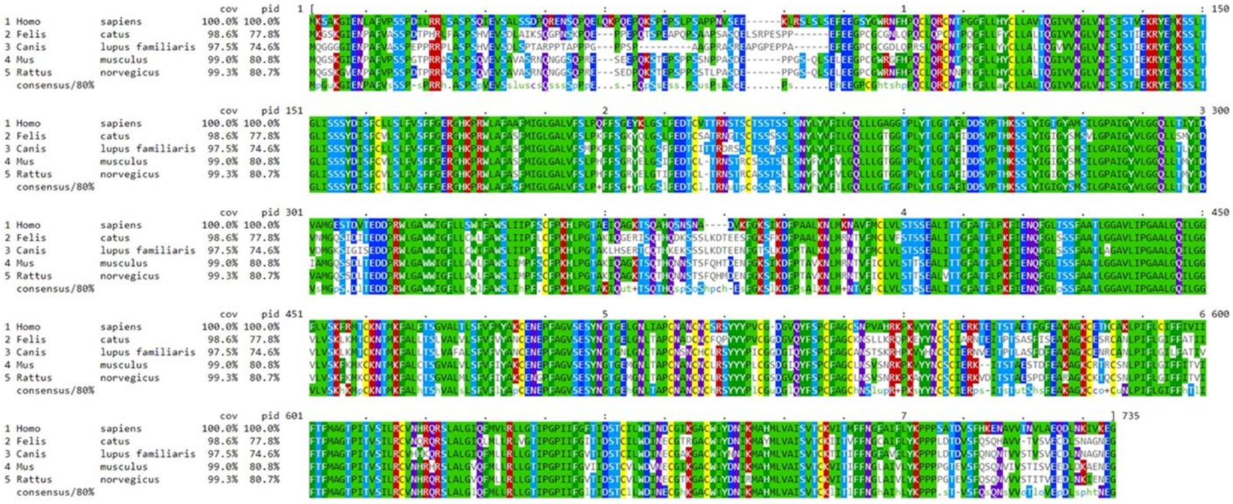

Supplementary Figure 3. Mammalian OATP4C1 protein sequence alignment. Mouse, rat, dog, and cat OATP4C1 protein sequences are compared to their human orthologous sequence. Cats share 77.8% OATP4C1 protein sequence identities with humans. Cov indicates the percentage of amino acids covered in the alignment, pid indicates protein sequence identity compared to humans, consensus denotes consensus out of 80% of the sequences from the group. Dog and cat OATP4C1 proteins are computationally predicted.

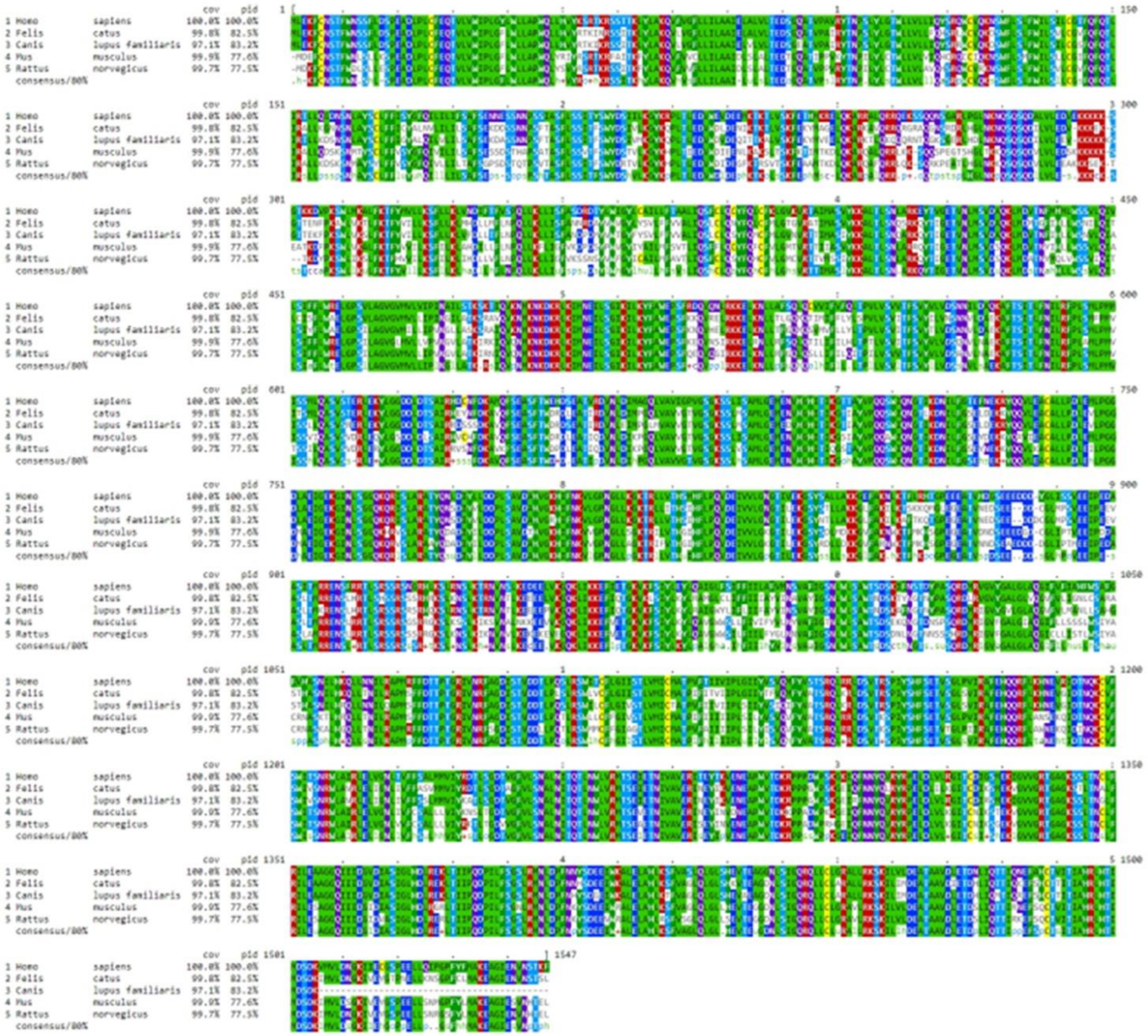

Supplementary Figure 4. Mammalian ABCC2 protein sequence alignment. Mouse, rat, dog, and cat ABCC2 protein sequences are compared to their human orthologous sequence. Cats share 82.5% ABCC2 protein sequence identities with humans. Cov indicates the percentage of amino acids covered in the alignment, pid indicates protein sequence identity compared to humans, consensus denotes consensus out of 80% of the sequences from the group. Dog and cat ABCC2 proteins are computationally predicted.

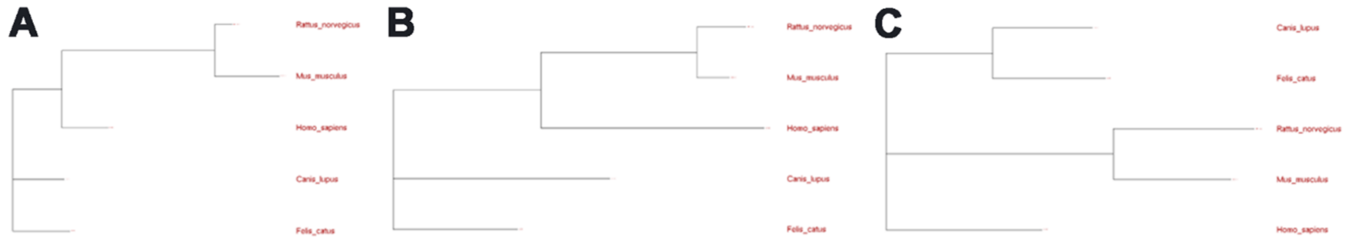

Supplementary Figure 5. Phylogenetic trees. A tree was constructed using (**A**) OAT1, (**B**) OATP4C1, and (**C**) ABCC2 protein sequences from humans, mice, rats, dogs and cats. Dogs and cats share a higher sequence similarity in all three proteins when compared with other mammalian species.
