# Supplementary material for: Impaired renal transporter gene expression and uremic toxin excretion as aging hallmarks in cats with naturally occurring chronic kidney disease
Source: Aging (Albany NY). 2024 Dec 20;16(22):13588–607. doi: 10.18632/aging.206176 (PMC11723653; doi:10.18632/aging.206176)
Supplement: Supplementary Table 1 [file aging-16-206176-s003.pdf]

## SUPPLEMENTARY TABLE

**Supplementary Table 1. Serum concentrations of metabolites in cohort 1.**

| | Mann whitney | Mean | | SEM | | Fold change |
|---|---|---|---|---|---|---|
| | P_MW | CON (ug/mL) | CKD2 (ug/mL) | SE_CON | SE_CKD2 | FC_CKD2vCON |
| TMAO | 0.00006 | 0.66 | 2.89 | 0.1 | 0.66 | 4.38 |
| IxS | 0.00067 | 1.19 | 4.79 | 0.25 | 1.19 | 4.03 |
| PCS | 0.00917 | 3.24 | 10.95 | 0.54 | 2.91 | 3.38 |
| PS | 0.00757 | 0.3 | 3 | 0.05 | 1.3 | 10 |
| IAA | 0.17651 | 0.49 | 0.59 | 0.12 | 0.14 | 1.2 |
| IPA | 0.27233 | 0.25 | 0.18 | 0.03 | 0.03 | 0.72 |
