# Supplementary material for: Impaired renal transporter gene expression and uremic toxin excretion as aging hallmarks in cats with naturally occurring chronic kidney disease
Source: Aging (Albany NY). 2024 Dec 20;16(22):13588–607. doi: 10.18632/aging.206176 (PMC11723653; doi:10.18632/aging.206176)
Supplement: Supplementary Table 3 [file aging-16-206176-s005.pdf]

Supplementary Table 3. Urine metabolites in cohort 1.

	Index to creatinine						Absolute concentration (ug/mL)	
	**P_MW**	**Mean_CON**	**Mean_CKD2**	**SEM_CON**	**SEM_CKD2**	**FC_CKD2_CON**	**Mean_CON**	**Mean_CKD2**
TMAO	0.00499	0.45	1.2	0.07	0.27	2.67	134.46	102.29
IxS	0.03377	0.69	1.5	0.12	0.31	2.17	199.81	150.46
PCS	0.03582	0.81	2.17	0.12	0.72	2.68	238.72	181.7
PS	0.16786	0.17	0.55	0.02	0.22	3.24	50	71.22
IAA	0.39102	0.02	0.01	0.01	0	0.5	6.62	1.16
