# Supplementary material for: Impaired renal transporter gene expression and uremic toxin excretion as aging hallmarks in cats with naturally occurring chronic kidney disease
Source: Aging (Albany NY). 2024 Dec 20;16(22):13588–607. doi: 10.18632/aging.206176 (PMC11723653; doi:10.18632/aging.206176)
Supplement: Supplementary Table 5 [file aging-16-206176-s007.pdf]

Supplementary Table 5. Serum metabolite concentrations of the healthy cats in cohort 1.

	YNG (15)	OLD (13)	FC	P-value
TMAO	0.44 ± 0.05	0.91 ± 0.19	2.1	0.036
IS	0.68 ± 0.09	1.78 ± 0.5	2.6	0.049
PCS	2.22 ± 0.45	4.59 ± 0.92	2.1	0.033
PS	0.24 ± 0.05	0.37 ± 0.08	1.5	0.20
IAA	0.41 ± 0.18	0.58 ± 0.16	1.4	0.50
IPA	0.27 ± 0.05	0.21 ± 0.04	0.8	0.36

The cats in the YNG group were less than 12 years old, while cats in the OLD group were 12 years or older. The numbers inside the parentheses indicate sample sizes. Data are expressed as mean ± SEM. *P*-values were from the Mann-Whitney test. Fold changes (FC) as the ratio of OLD/YNG. TMAO, rimethylamine N-oxide; IS, indoxyl sulfate; PCS, p-cresol sulfate, PS, phenyl sulfate; IAA, indole-3 acetic cid: IPA, indole-3 propionic acid.
