# Supplementary material for: Impaired renal transporter gene expression and uremic toxin excretion as aging hallmarks in cats with naturally occurring chronic kidney disease
Source: Aging (Albany NY). 2024 Dec 20;16(22):13588–607. doi: 10.18632/aging.206176 (PMC11723653; doi:10.18632/aging.206176)
Supplement: Supplementary Table 7 [file aging-16-206176-s009.pdf]

Supplementary Table 7a. Renal cortical tissue samples.

	Control	CKD1/2	CKD3/4	Amyloid	P-value
N = 24	6	8	7	3	
Age (yrs)	13.7 + 1.04	14.2 + 0.91	12.8 + 1.11	14.6 + 0.57	0.92
Sex (F/M)	4/2	4/4	4/3	3/2	0.95

Supplementary Table 7b. Renal medulla tissue samples.

	Control	CKD1/2	CKD3/4	Amyloid	P-value
N = 21	6	7	5	3	
Age (yrs)	13.7 + 1.16	13.8 + 1.09	12.2 + 1.29	13.3 + 0.66	0.95
Sex (F/M)	4/2	3/4	3/2	3/0	0.48
